# Antibacterial and Disinfecting Effects of Standardised Tea Extracts on More than 100 Clinical Isolates of Methicillin-Resistant *Staphylococcus aureus*

**DOI:** 10.3390/plants12193440

**Published:** 2023-09-29

**Authors:** Ruth Feilcke, Volker Bär, Constanze Wendt, Peter Imming

**Affiliations:** 1 Institut für Pharmazie, Martin-Luther-Universität Halle-Wittenberg, Kurt-Mothes-Str. 3, 06120 Halle, Germany; 2 Zentrum für Infektiologie, Universität Heidelberg, Im Neuenheimer Feld 324, 69120 Heidelberg, Germany; 3 Labor Dr. Limbach & Kollegen GbR, Medizinisches Versorgungszentrum, Im Breitspiel 15, 69126 Heidelberg, Germany

**Keywords:** multi-resistance, *Staphylococcus*, tea extracts, *Camellia sinensis*, time–kill plot

## Abstract

Methicillin-resistant *Staphylococcus aureus* (MRSA) infections are still a major problem in hospitals. The excellent safety profile, accessibility and anti-infective activity of tea extracts make them promising agents for the treatment of infected wounds. To investigate the possibility of sterilising MRSA-infected surfaces, including skin with tea extracts, we determined the MICs for different extracts from green and black tea (*Camellia sinensis*), including epigallocatechin gallate (EGCG), on a large number of clinical isolates of MRSA, selected to represent a high genetic diversity. The extracts were prepared to achieve the maximal extraction of EGCG from tea and were used as stable lyophilisate with a defined EGCG content. All extracts showed a complete inhibition of cell growth at a concentration of approx. 80 µg/mL of EGCG after a contact time of 24 h. Time–kill plots were recorded for the extract with the highest amount of EGCG. The reduction factor (RF) was 5 after a contact time of 240 min. EGCG and tea extracts showed an RF of 2 in methicillin-sensitive *S. aureus.* Extracts from green and black tea showed lower MICs than an aqueous solution with the same concentration of pure EGCG. To the best of our knowledge, we are the first to show a reduction of 99.999% of clinically isolated MRSA by green tea extract within 4 h.

## 1. Introduction

Infections with methicillin-resistant *Staphylococcus aureus* (MRSA) have become a large problem for hospitals in most countries since 1990. Together with cephalosporin-resistant *Escherichia coli*, MRSA still causes the highest portion of disability-adjusted life years by infections with antibiotic-resistant bacteria in Europe [1] and is the second of six leading pathogens for deaths associated with resistance worldwide [2]. Within Europe, the portion of MRSA in invasive isolates shows a high variability between different countries, ranging from 0.9% in Norway to 42.9% in Cyprus in the years 2020–2022 [3,4]. Although the EU/EEA population-weighted MRSA percentage has been decreasing for more than 5 years now, the treatment of MRSA infections is still a major issue as they appear to cause a higher mortality rate than those caused by methicillin-susceptible *S. aureus* [5].

The antimicrobial activity of green and black tea extracts (leaves of *Camellia sinensis*) and their isolated constituents has been known since 1906 [6]. In vitro data indicated that tea extracts of ordinary brewing strength also inhibited the growth of MRSA [7]. Of the tea extract constituents, epigallocatechin gallate (EGCG) was especially investigated and was reported to have an MIC against *S. aureus* below 100 µg/mL [8]. In vivo studies on the activity of tea extracts against MRSA are rare but provide reason for hope [9,10,11]. Although the antibacterial and antiviral effects, excellent safety profile and easy accessibility of tea extracts are well-known, their routine and controlled application in medicine is still uncommon. To guide clinicians on how to apply tea extracts, we set out to investigate their practicality as disinfectants. In contrast to published reports, we set on preparing extracts with high amounts of EGCG along with low amounts of caffeine, and standardised the extracts for EGCG.

The purpose of this study was to investigate the activity of different *Camellia sinensis* extracts with defined EGCG content against a large number of genetically diverse MRSA strains that were isolated in hospitals in Germany [12] compared with pure EGCG as the lead compound and positive control, by determining minimum inhibitory concentrations, and—very important for sterilisation and disinfection—by determining the contact time that led to a significant reduction in colony-forming units. All steps were optimised as for practical handling to serve as a guideline for the facile preparation of solutions for the topical treatment of MRSA infections.

## 2. Results

### 2.1. EGCG Content of the Tea Extracts

While Wang et al. (2008) [13] wrote that EGCG epimerised rapidly but was only slowly hydrolysed by high temperatures and boiling water, we found that EGCG easily hydrolyses and underlies oxidative processes in solution. For the purpose of this study, we therefore prepared a storage-stable lyophilizate, which would also be suitable for antibacterial treatment after dissolving in water. A lyophilised green tea extract (GTE) and black tea extract (BTE) were examined for EGCG content using the method described in Section 4.2. The EGCG content of GTE (20%) was twice the amount of BTE (11%). Consequently, the green tea extract was diluted to obtain standardised solutions of equal EGCG content (see Section 4.3.1 and Section 4.3.2).

### 2.2. Comparison of Inhibition by Pure EGCG, Black and Green Tea Extracts in Selected MRSA Strains

**Figure 1:**
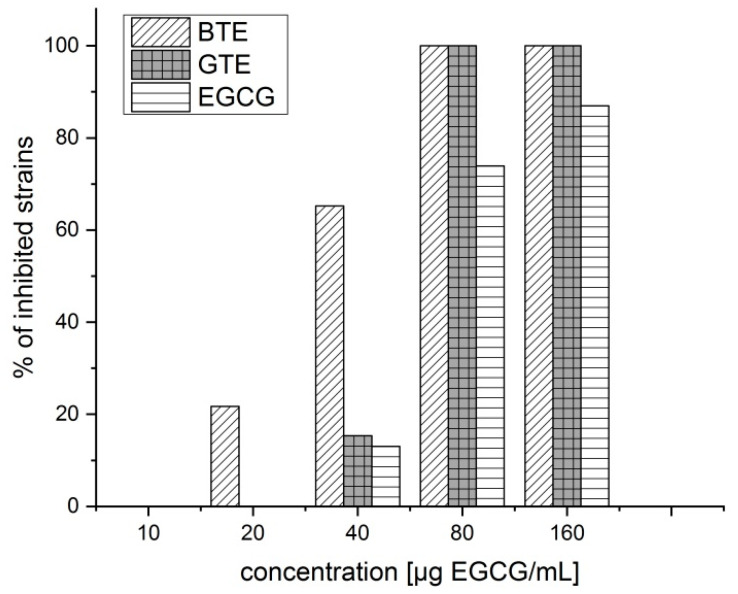
Comparison of the inhibition effectivity of different tea extracts in comparison with pure EGCG on MRSA. GTE was tested on 111 strains, while BTE and EGCG on 23 different strains (clinical isolates and type cultures, see Section 4.3.3). BTE, black tea extract; GTE, green tea extract; MRSA, methicillin-resistant *Staphylococcus aureus*.

The effect of different tea extracts and EGCG on the bacterial growth of 23 different strains is shown in Figure 1. At 40 µg/mL EGCG content, BTE inhibited more than 60% of the tested strains, whereas pure EGCG and GTE inhibited approx. 10% only. Table 1, “Average”, shows that there is no statistically significant difference in MIC between BTE, GTE and EGCG. However, this satisfies the fact that there are differences in sensitivity between individual strains. Since in medical practice, strain identification are usually not performed, mixtures of BTE and GTE should be used.

### 2.3. MIC50 and MIC90

**Figure 2:**
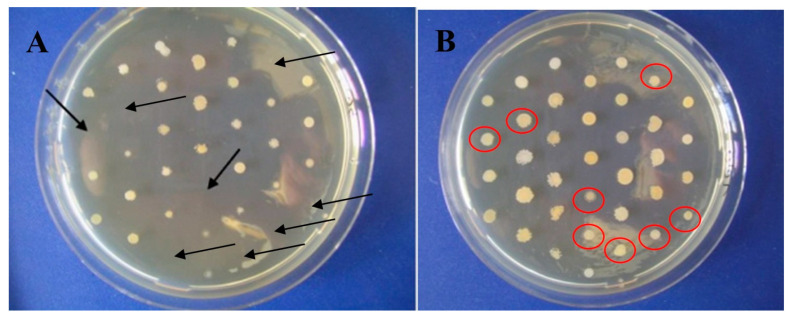
Comparison of the inhibitory effect of GTE on a selection of 36 different MRSA strains. (**A**) Colonies at 40 µg/mL EGCG in GTE. (**B**) Colonies at 20 µg/mL EGCG in GTE. The arrows highlight the absence of growth, the red circles show the same strains growing at the lower concentrations of EGCG. EGCG, epigallocatechin gallate; GTE, green tea extract; MRSA, methicillin-resistant *Staphylococcus aureus*.

MIC50 as well as MIC90 were determined for the different solutions. The MIC50 and MIC90 of the GTE were determined to be 80 µg EGCG/mL each. However, inhibitory effects became visible at lower concentrations (Figure 2 and Table 1). 

The average MIC for GTE out of all 111 tested strains was 74 ± 14 µg EGCG/mL. The MIC50 and MIC90 of the BTE, expressed as EGCG content, were determined to be 40 and 80 µg EGCG/mL, respectively. Pure EGCG had a weaker antibacterial activity compared to both tea extracts with MIC50 and MIC90 at 80 and 160 µg EGCG/mL, respectively. Table 1 lists MICs for a number of tested strains. The average MIC (49 µg EGCG/mL) of BTE out of 23 strains tested on both extracts was lower than that of GTE (60 µg EGCG/mL). Only three strains were less sensitive for BTE than for GTE. In more than half of the strains (13/23), BTE was more active than GTE.

### 2.4. Effect of Contact Time on Reduction Factor

**Figure 3:**
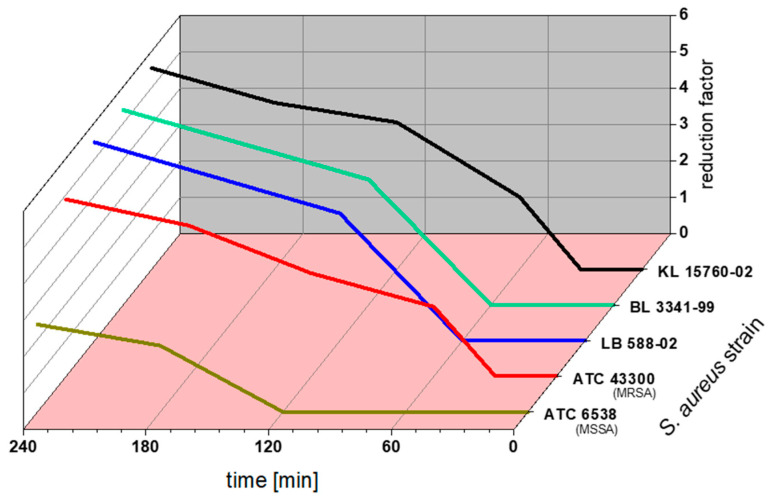
Three-dimensional plot of contact time, reduction factor and *S***.** *aureus* strain for GTE at 80 µg EGCG/mL. EGCG, epigallocatechin gallate; GTE, green tea extract.

Figure 3 shows the dependency of the reduction factor of GTE at 80 µg EGCG/mL on contact time for selected bacterial strains. All strains showed a higher reduction with time elapsing. The contact time required to kill 99.999% of the bacteria depended on the tested strains. We found a higher sensitivity for the GTE of clinical isolates than of the strains from culture collections.

Although ATCC 43300 showed the first effects after 60 min, ATCC 43300 and ATCC 6538 did not reach the intended reduction factor of 5, which all clinical isolates reached within 4 h. However, we did not see a concentration-dependant fashion as the tested concentrations of 40, 80 and 160 µg EGCG/mL of the GTE showed similar results (see Figure S1).

## 3. Discussion

Usual brewing techniques normally yield concentrations of 10–45 µg EGCG /mL for black tea and 78–130 µg EGCG/mL for green tea [14]. By preparing a lyophilizate, we achieved a higher EGCG content that allowed us to maintain this concentration range even after dilution with agar. At the same time, this lyophilisate is chemically much more stable and can easily be dissolved in water.

EGCG was chosen as the lead and standard because: (1) it is present in all kinds of tea (fermented, semi fermented and non-fermented) in low to high amounts [14,15,16,17] and (2) of all antibacterial constituents, EGCG was found to have the lowest MIC [18]. EGCG uniquely enables a direct activity comparison of black and green tea extracts. 

The tested solutions were all active against clinically isolated MRSA as well as against strains from culture collections of MSSA and MRSA. There were differences in susceptibility of the different strains, as expected, because of genetic diversity. Clinical isolates showed a higher susceptibility than type cultures. While the precise reason for this was not investigated, it lends support to the suggested use of tea extracts against MRSA infections. Solutions containing only pure EGCG showed the lowest activity, necessitating higher concentrations for growth inhibition. This corroborates that antibacterial activity in tea extracts is not only based on EGCG, even though this compound is the one with the highest activity among the constituents investigated in the past [19]. BTE was more active than GTE, confirming the finding of Yam et al. [20], who reported that out of 17 teas (black, green and semi-fermented), a black tea infusion showed the highest antimicrobial potency. The different antimicrobial activities of different preparations of *Camellia sinensis* leaf extracts most likely are due to the additional presence of mono-, oligo- and polyphenols (e.g., EGC, ECG, EG). Theaflavin and epicatechin gallate (EG), for instance, were shown to exhibit antimicrobial properties [18]. They occur at comparatively high concentrations in BTE and GTE [18]. However, it needs to be underscored that the solution of BTE needed to be about twice as concentrated as the solution of the GTE in order to adjust the content of EGCG. This consequently led to higher concentrations of additional polyphenols in BTE and is most probably the reason for the higher antibacterial activity of BTE in our study.

Yam et al. [20] presented killing plots over time for a type of Japanese green tea. They only used the strain *S. aureus* USA 12 in 1997, unlike our study, which used a large number of strains selected from clinical isolates and laboratory strains. Yam et al. [20] used a 2% solution, while we used 3.75%. On this basis, the MIC of 0.28 mg tea extract/mL which they reported is comparable to the 30 µg EGCG/mL in this work, although the average MIC of our extract was 60 µg EGCG/ ml. Therefore, while their green tea extract had half the MIC as that which was reported by us, their extract needed three times longer [20] to reduce the number of bacteria than our extract. The first effects were only seen after 8 h at a concentration of 1.5 mg/mL of a 2% tea extract. This concentration is comparable to 160 µg EGCG/mL of the green tea extract in our study that showed a reduction of 99.9% in bacteria after 4 h in four out of five strains (strains from collections and clinical isolates; see Figure S2). Zihadi et al. found a minimum bactericidal concentration (MBC) for green tea extract of 31 mg/mL [21]. In our study, the GTE of 0.4 mg/mL (containing 80 µg EGCG/mL) already led to bactericidal effects, determined as reduction in CFUs. The differences very likely resulted from differences of the tested strain(s). Differences in the amount of constituents in the green tea may also have caused a lower effect of their extract. We could not confirm the concentration dependence reported by Yam et al. as all three tested concentrations in this work showed similar results (see Figures S1 and S3). But our study clearly shows that it is possible to reduce the number of bacteria by a factor of log 5 with an extract from green tea within 4 h, since all clinical isolates tested showed a reduction factor of 5. This establishes that extracts from tea not only have bacteriostatic capabilities, but also bactericidal effects. However, even though these results are promising, the time needed for eradication is too long for its usage as a proper disinfectant.

Based on our results and the literature data, some applications for tea extracts in MRSA infections include: (1) the topical treatment of MRSA infections (skin, oral mucosa); and (2) the decolonisation treatment of MRSA-positive patients before surgery. This presently relies on mupirocin, chlorhexanide, octenidine or triclosan [22,23], which have considerably lower MICs [24] than tea extracts. However, in contrast to chlorhexidine and other standard disinfectants, *Camellia sinensis* extracts do not inhibit cell proliferation and there are also no known microbial resistances to this class of substances; whereas in the last years, concerns about resistance of MRSA against several common disinfectants arose [25,26,27]. Systemic MRSA infections cannot be treated with tea extracts and catechins because the concentrations of EGCG approaching the growth inhibitory concentrations found in this study will not be reached after an oral application of up to 800 mg of EGCG [28,29,30]. Thus, taken together, for the treatment of large wounds, e.g., after massive burns, and for long-term treatment, a well-tolerated antibacterial with no resistance such as BTE and GTE would be an asset. Treatment should employ mixtures of BTE and GTE as we found different sensitivities of *S. aureus* strains towards either BTE or GTE.

Another possible application consists of the inhalation of aqueous solutions of tea extracts—the same way isotonic sodium chloride solutions are widely used for common colds and dry airways, especially in ventilator bound patients, which requires moisture in the air. The first clinical data in this field provide reason for hope [9], and the extraction process in this work by using 90 °C water instead of boiling water leads to a lower caffeine content [17]. 

To the best of our knowledge, we are the first to show a reduction of 99.999% of clinically isolated MRSA by green tea extract within 4 h. Additionally, our results suggest that MRSA is more susceptible to BTE and EGCG than MSSA. Although statistical significance cannot be proven, Si et al. (2006) [19] showed comparable results, while Cho et al. reported similar MICs for green tea extract against MSSA and MRSA [31].

## 4. Materials and Methods

### 4.1. Preparation of Lyophilisates

The plant material was obtained from the local-state food inspection authority, where its identity had been ascertained beforehand. The plant name was checked with http://www.theplantlist.org. Different tea samples were subjected to HPLC to determine the content of EGCG, and teas with high content were chosen for the preparation of lyophilisates. A total of 1.5 g of dry tea samples was crushed and extracted for 10 min in 40 mL of water at 90 °C. Following filtration and cooling to room temperature, the extract was cleared from solids by centrifuging at 5000 rpm. The clear extract was frozen and freeze-dried. We obtained 0.8 g of lyophilisate that was stored in a desiccator at room temperature. 

### 4.2. Determination of EGCG Content in Lyophilisates by Quantitative Thin-Layer Chromatography

The lyophilisate (10 mg) was suspended in 5 mL of methanol, centrifuged, and the supernatant subjected to quantitative HPTLC. The procedure followed a published method [32] with changes in mobile phase and detection as described: mobile phase—chloroform, ethyl acetate, methanol, toluene and formic acid (12:8:4:4:2); detection—scanning with a dual wavelength TLC-scanner type CS-930 from Shimadzu (Kyoto, Japan) at a wavelength of 284 nm directly after drying for 10 min. The reference was 99.36% pure EGCG (PhytoLab, Vestenbergsgreuth, Germany).

### 4.3. Antibacterial Assays

#### 4.3.1. Lyophilisates and Solutions Tested

##### Green Tea Extract

An amount of 800 mg of GTE lyophilisate (containing 160 mg of EGCG) was dissolved in 50 mL purified water and 355 mg of ascorbic acid was added for stabilisation as suggested by Hatano et al. [33]. We chose a molar ratio of EGCG to ascorbic acid of 5.76 because tea extracts contain a mixture of catechins that are all stabilised by ascorbic acid. This parent solution was diluted by adding purified water to yield solutions of GTE with 1.6 mg EGCG/mL, 0.8 mg EGCG/mL, 0.4 mg EGCG/mL, 0.2 mg EGCG/mL and 0.1 mg EGCG/mL.

##### Black Tea Extract

An amount of 160 mg of BTE lyophilisate (containing 16 mg of EGCG) and 35.5 mg of ascorbic acid (molar ratio of EGCG to ascorbic acid: 5.76) were dissolved in 10 mL of purified water and further diluted by adding purified water to yield solutions of BTE with the same EGCG concentrations as the GTE.

##### Pure EGCG

An amount of 16.26 mg of EGCG (purity 98.4%) and 17 mg of ascorbic acid (molar ratio of EGCG to ascorbic acid of 2.8 due to absence of additionally catechins) were dissolved in 10 mL purified water and diluted by adding purified water to obtain the same concentrations of EGCG as in the tea extracts.

#### 4.3.2. Preparation of Agar Plates

The preparation followed the standard protocol in [34] for the “agar dilution method for antimicrobial testing” with the following specific procedure. An aliquot of 2 mL of the appropriate dilution of extract was filled up to 20 mL with standard Mueller–Hinton agar at 55 °C, yielding test concentrations from 20 to 160 µg/mL EGCG equal to 0.002–0.016% EGCG (see Table 2). The plates were carefully tilted to ensure mixing. Four plates with GTE and one plate of BTE and pure EGCG were prepared. Additionally, one plate was poured for each dilution only containing the corresponding amount of ascorbic acid as negative control. The plates were stored at 6 °C until use.

#### 4.3.3. Selection of Strains for Determining the MIC of Tea Extracts and Pure EGCG

The strains used in this study were collected during a longitudinal study in the university hospital of Heidelberg, Germany [12]. In summary, the strains listed in Table 3 were used for MIC determination. As clinical isolates of MRSA are often clones of strains isolated previously, we purposefully looked for high genetic diversity.

GTE showed similar results for the 111 strains tested. Considering time and economy factors, we reduced the number of strains tested with BTE and pure EGCG to a limited amount of strains (10 strains with MIC_GTE_ 40 µg EGCG/mL, 10 strains with MIC_GTE_ 80 µg EGCG/mL and 3 reference strains). Table 1 lists these strains. 

#### 4.3.4. Cultivation of Inocula and Inoculation of Agar Plates

The procedure followed the protocol “growth method” described in [34]. The exact steps were as follows. All 111 MRSA-strains were cultivated on blood agar plates (blood agar Columbia, 5% sheep blood, obtained from Oxoid Ltd., Hampshire, United Kingdom). The cultures were individually transferred, using an inoculating loop, and inoculated on plates consisting of the same agar and incubated at 36 °C for 48 h. After visually checking for pure cultures, a culture was taken with a one-time inoculating loop and put into a test tube filled with 10 mL of Mueller–Hinton–Bouillon. The tube was then blanked off and incubated for another 24 h at 36 °C. For each tube, 1 mL of incubated bacteria suspension was taken and mixed with 10 mL of 0.9% NaCl solution. This suspension was checked photometrically and inoculum was prepared to be 1 × 10^8^ CFU/mL. Inoculation of prepared agar plates (Section 4.3.2) was carried out using a multipoint inoculator with 750 µL of bacteria suspended in 0.9% NaCl solution. A maximum of 36 MRSA strains was cultivated per agar plate at 36 °C for 24 h.

#### 4.3.5. Logging of Bacterial Growth; MIC Determination

The agar plates were visually checked for growth. Distinct growth was noted as “+” and less than 10 colonies was noted as “−”. MIC was determined as the lowest concentration where “−” was noted. MIC50 and MIC90 were set as the concentrations at which a growth of 50% or 90%, respectively, of all tested strains were inhibited. 

#### 4.3.6. Determination of Reduction Factor of Different Green Tea Extracts

The tests were conducted using the European Standard Method for bactericidal activity in the medical area [35]. The method includes ‘organic stress’, which is intended to simulate biological conditions. Since GTE was examined using this method for the first time, with no data for comparison available, we abstained from applying ‘organic stress’. The test solution was prepared freshly by dissolving 800 mg of GTE with an EGCG content of 20% in 50 mL of water for injection (Ph.Eur.) and adding 35.5 mg of ascorbic acid. This parent solution had an EGCG content of 3.2 mg/mL. The required test solutions were prepared by diluting the parent solution with water for injection to 200, 100 and 50 µg EGCG/mL, respectively. A total volume of 8 mL of the testing solution was mixed with 1 mL purified water and 1 mL of inoculum to result in a starting an inoculation concentration of 10^9^ CFU/mL because we tested the extracts as disinfectants, not as antibiotics. The mixture was incubated at 36 °C and 500 µL samples were taken after 30, 60, 120, 180 and 240 min. They were neutralised by dilution with 4.5 mL of a neutralising solution (polysorbate 80 30 g/L, lecithin 3 g/L, l-cystein 1 g/L, aqua bidest. ad 1000 mL) and well mixed. After 5 min, dilutions of this mixture were prepared 10 and 100 times and plated on agar to be incubated at 36 °C for 42 h for the counting of CFUs. Two control samples were measured to exclude an influence using the neutralisation method, and two control samples without tea extract were measured to determine the CFU without inhibitory effect (CFUx).

The efficacy of growth inhibition was assessed by plotting the contact time of the three GTE concentrations against the reduction factor. The reduction factor was calculated using this formula: lg(RF) = lg(CFUx) − lg(CFUy), where CFUx is the number of CFU without the addition of GTE and CFUy is the number of CFU after exposure to a certain concentration of GTE for a specified time.

## 5. Conclusions

Our study proves that it is easy to prepare aqueous solutions of tea extracts that reach the MICs we found. For a tea that contains 10% of EGCG, 160 g will yield a concentration of 80 µg/mL EGCG in 200 L of water (a bathtub). This is the concentration that inhibits the growth of all MRSA strains tested. In order to reach the satisfactory reduction factor of at least 5, according to our data, the bacteria need to be in contact with the solution for at least four hours (see Section 2.4, Figure 3). Consequently, the potential application of black or green tea extracts and EGCG may aid in the long-term treatment of chronical lesions, including the prevention of MRSA (re-)colonisation. We suggest a solution or hydrocolloid dressing, for example, on the basis of carboxymethyl cellulose, containing tea extract that would easily achieve the exposure time necessary for a log 5 reduction. It combines easy preparation with good tolerability. 

Prospectively, tea extracts might help to fight the increasing resistance rate by reducing the amount of applied reserve antibiotics. Because of the necessary contact time of 4 h and more, an exclusive tea extract treatment is not very promising, but as an addition to standard therapy, it could prove to be a cost-efficient way of ensuring complete eradication. In some instances, the slightly astringent side-effect may indeed help with treatment, e.g., soft-tissue healing.

## Figures and Tables

**Table 1:** Comparison of activity of GTE, BTE and pure EGCG on the growth of MRSA strains. MIC expressed in µg/mL of EGCG content on 23 different strains. BTE, black tea extract; EGCG, epigallocatechin gallate; GTE, green tea extract; MIC, minimal inhibitory concentration; MRSA, methicillin-resistant *Staphylococcus aureus;* MSSA, methicillin-sensitive *Staphylococcus aureus*.

Strain	BTE	GTE	Pure EGCG
BL12357-98	20	40	40
BL14179-99	20	40	80
BL3341-99	80	80	80
BL5024-99	80	80	160
BL5808-02	40	40	80
BL9783-98	80	40	80
CH18203-97	20	40	80
HY138-00	20	40	40
HY1971-02	40	80	80
HY1975-02	40	80	160
HY2075-03	80	40	160
HY684-01	80	40	80
KL11072-00	20	40	40
KL14292-00	40	80	80
KL1486-00	40	80	80
KL15760-02	40	80	80
KL2495-99	80	80	80
LB2301-99	80	80	80
LB5375-02	40	80	>160
LB5714-03	40	40	80
Average	49 ± 25	60 ± 21	88 ± 39
MIC 50	40	80	80
MIC 90	80	80	160
MRSA ATCC 43300	40	80	80
MSSA ATCC 29213	80	80	160
MSSA ATCC 6538	40	80	>160

**Table 2:** EGCG solutions and extracts used in this study. Column 1 and 2 show the percentage concentration of the whole extracts. Column 3 and 4 list the EGCG content in µg/mL% in the extracts. EGCG: Epigallocatechin gallate; GTE: Green tea extract; BTE: Black tea extract.

Concentration	GTE Extract (%)	BTE Extract (%)	EGCG in Extract (µg/mL)	EGCG in Extract (%)
Dilution 1	0.08	0.16	160	0.016
Dilution 2	0.04	0.08	80	0.008
Dilution 3	0.02	0.04	40	0.004
Dilution 4	0.01	0.02	20	0.002

**Table 3:** Number of *Staphylococcus aureus* strains used for MIC determination. BTE, black tea extract; GTE, green tea extract; MRSA, methicillin-resistant *Staphylococcus aureus*; MSSA, methicillin-sensitive *Staphylococcus aureus*.

Strain	GTE	BTE
Clinical MRSA isolates	108	20
ATCC-MRSA strains	1	1
ATCC-MSSA strains	2	2

## Data Availability

The data presented in this study are available on request from the corresponding author.

## Supplementary Materials

The following supporting information can be downloaded at: https://www.mdpi.com/article/10.3390/plants12193440/s1, Figure S1. Effect of GTE on reduction factor of MSSA ATCC 43300 at tested concentrations. Figure S2. Time-kill-plot for GTE with 160 µg/mL EGCG content for the tested strains. Figure S3. Overview of results for the time-kill-plot experiment.

## Abbreviations

BTE: black tea extract; EG: epicatechin gallate; EGC: epigallocatechin; EGCG: epigallocatechin gallate; GTE: green tea extract; Ph.Eur.: European Pharmacopoeia; RF: reduction factor; MIC: minimal inhibitory concentration; MRSA: methicillin-resistant *Staphylococcus aureus*; MSSA: methicillin-sensitive *Staphylococcus aureus*.
