# Supplementary material for: Antibacterial and Disinfecting Effects of Standardised Tea Extracts on More than 100 Clinical Isolates of Methicillin-Resistant Staphylococcus aureus
Source: Plants (Basel). 2023 Sep 29;12(19):3440. doi: 10.3390/plants12193440 (PMC10575227; doi:10.3390/plants12193440)
Supplement: Supplementary file 1 [file plants-12-03440-s001.zip › plants-2577106-supplementary.pdf]

Supplementary Materials

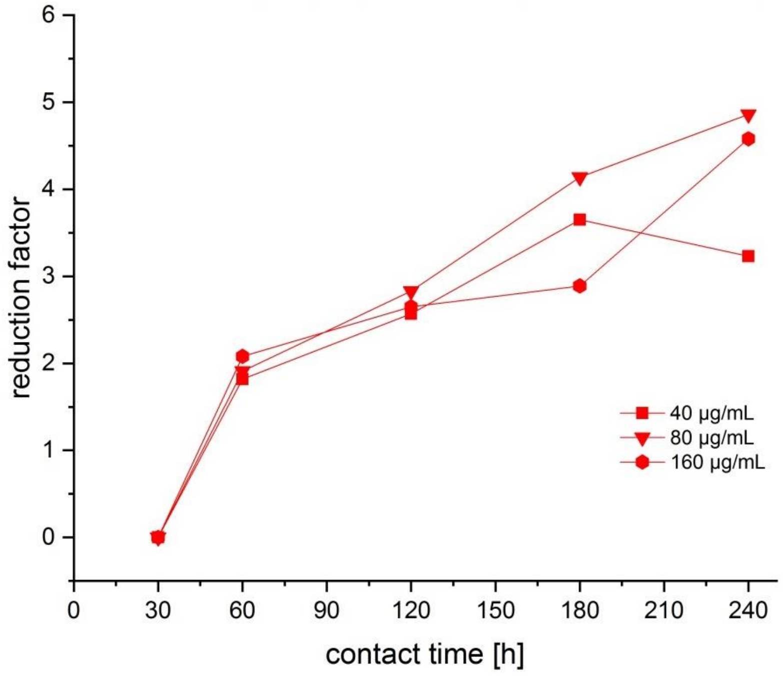

Figure S1. Effect of GTE on reduction factor of MSSA ATCC 43300 at tested concentrations.

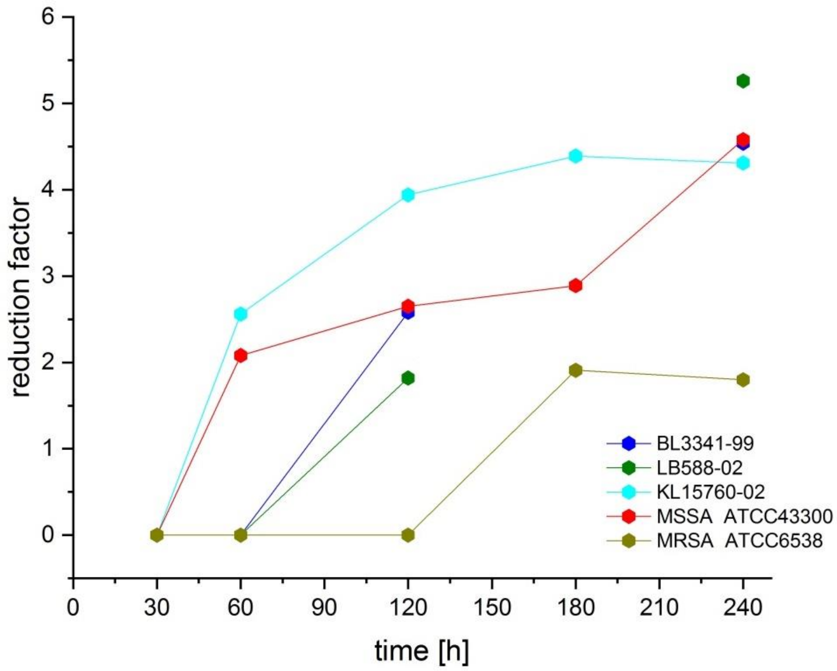

Figure S2. Time-kill-plot for GTE with 160 µg/mL EGCG content for the tested strains.

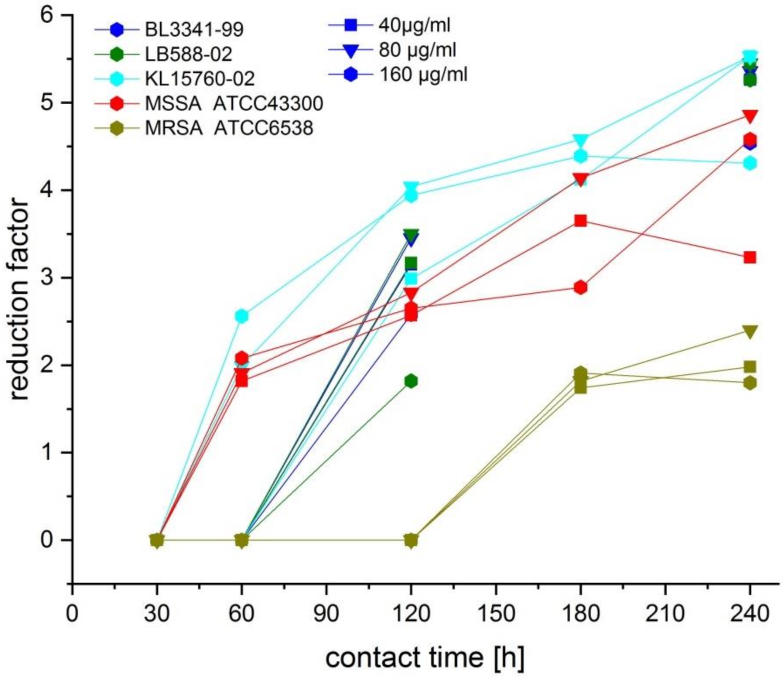

Figure S3. Overview of results for the time-kill-plot experiment.
